# Genetic and genomic variability of *Spiroplasma* and *Midichloria* endosymbionts associated with the tick *Ixodes frontalis*

**DOI:** 10.1093/ismeco/ycaf202

**Published:** 2025-11-10

**Authors:** Sophie Melis, Leandro Gammuto, Michele Castelli, Tiago Nardi, Beatrice Bisaglia, Olivier Duron, Alessandra Cafiso, Julie Botman, Olivier Lambert, Emanuela Olivieri, Hein Sprong, Olivier Plantard, Davide Sassera

**Affiliations:** Department of Biology and Biotechnology, University of Pavia, 27100, Pavia, Italy; Department of Biology and Biotechnology, University of Pavia, 27100, Pavia, Italy; Department of Biology and Biotechnology, University of Pavia, 27100, Pavia, Italy; Department of Biology and Biotechnology, University of Pavia, 27100, Pavia, Italy; Department of Clinical Sciences and Community Health, Dipartimento di Eccellenza 2023-2027, University of Milan, 20133, Milan, Italy; Department of Biology and Biotechnology, University of Pavia, 27100, Pavia, Italy; Centre of Applied Studies for the Sustainable Management and Protection of Mountain Areas (CRC Ge.S.Di.Mont.), University of Milan, 25048, Edolo, BS, Italy; MIVEGEC (Maladies Infectieuses et Vecteurs: Ecologie, Génétique, Evolution et Contrôle), University of Montpellier (UM), Centre National de la Recherche Scientifique (CNRS), Institut pour la Recherche et le Développement (IRD), Montpellier 34394, France; Department of Veterinary Medicine and Animal Sciences, University of Milan, 26900, Lodi, Italy; Centre Vétérinaire de la Faune Sauvage et des Écosystèmes, Oniris, CHUV, Nantes 44300, France; Centre Vétérinaire de la Faune Sauvage et des Écosystèmes, Oniris, CHUV, Nantes 44300, France; Istituto Zooprofilattico Sperimentale della Lombardia e dell’Emilia Romagna, 27100, Pavia, Italy; Centre for Infectious Disease Control, National Institute for Public Health and the Environment, 3720 BA, Bilthoven, the Netherlands; Oniris, INRAE, BIOEPAR, 44307, Nantes, France; Department of Biology and Biotechnology, University of Pavia, 27100, Pavia, Italy; Fondazione IRCCS Policlinico San Matteo, 27100, Pavia, Italy

**Keywords:** *I. frontalis*, symbiont, genomics, *Midichloria*, *Spiroplasma*

## Abstract

*Ixodes frontalis*, an ornithophilic tick species, is widely distributed all over Europe exhibiting two genetically diverging haplogroups based on differences in the cytochrome *c* oxidase subunit 1 mitochondrial gene. Despite its broad distribution, little is known about the presence of symbiotic bacteria in *I. frontalis*, while symbionts are generally widespread in ixodid ticks and responsible for important effects on host fitness. We collected *I. frontalis* from France and Italy (*n =* 277) and assessed that the most prevalent haplogroup was A (73%). We then investigated the presence of the symbionts, *Midichloria mitochondrii* and *Spiroplasma ixodetis*. They were both found at a high prevalence in adult ticks (66% and 77% respectively), while the number of positive immature ticks was significantly lower (18% for both). The experimental analysis of larvae hatched from egg clutches obtained from four females hints at vertical transmission of both symbionts. We obtained three genomes of *Spiroplasma* and one of *Midichloria,* and used them to perform comparative genomic analysis. Average nucleotide identity among available *Spiroplasma* or *Midichloria* genomes from *I. frontalis* are all extremely high*,* suggesting low genetic variability for both symbionts. Gene presence/absence analysis confirmed the presence of B vitamin synthesis genes in the genome of *M. mitochondrii*, and also showed the presence of the ETX/MTX2 gene, the RIP family and a partial Spaid-like gene in *S. ixodetis*. This gene repertoire indicates a nutritional role for *Midichloria*, while for *S. ixodetis* we hypothesize a role of this bacterium as a defensive symbiont or a manipulator of the host reproduction.

## Introduction

*I. frontalis* is an ornithophilic hard tick species widespread in Europe that belongs to the family Ixodidae [1, 2]. Ticks of this species have been found mainly on birds, especially on passerines [3] While reports of parasitism on humans are rare [4–6] *I. frontalis* is responsible for the poorly understood avian-tick related syndrome [7] and it is known to harbor bacterial pathogens such as *Neoehrlichia mikurensis* [8], *Anaplasma phagocytophilum* [9], *Rickettsia* spp. [10], and *Borrelia burgdorferi* sensu lato [11]. Knowledge on its vectorial competence is however still limited [12]. Two divergent mitochondrial haplogroups of *I. frontalis* (named A and B) have been identified based on differences in cytochrome *c* oxidase I (COI) and 16S genes, leading to the hypothesis that they may correspond to two cryptic species [13, 14]. However, no morphological differences were observed between nymphs of the two haplogroups [15]. Available data about the respective prevalences or geographical distribution of those two haplogroups remains scarce [13, 15, 16].

The obligate hematophagous lifestyle of ticks, which rely solely on blood meal as a source of energy, favours contact and potential associations with many bacterial species. These associations may evolve towards mutualism, with the symbionts exerting one or multiple diverse positive effects on tick fitness by contributing to various physiological processes, including nutrition, development and reproduction [17, 18]. The most common bacterial mutualists in ticks belong to the gammaproteobacterial genera *Francisella* and *Coxiella*, which have been proved to be essential for development and moult in multiple tick species, providing B vitamins, in particular biotin, riboflavin and folate, and the removal of the bacteria leads to severe detrimental effects [19–22]. For instance, *Francisella* endosymbionts have been shown to provide their host, the soft ticks *Ornithodoros moubata,* with B vitamins that are lacking in the blood diet [23]. Consistently with a required role for the respective hosts, such symbionts were reported to have an almost full prevalence and high abundance in adult females in multiple host populations [24, 25]. On the other hand, immature stages and adult males tend to have lower and hardly detectable symbiont loads, particularly after prolonged starvation, while they generally increase after blood meals. In any case, a pattern of high prevalence of a single bacterial symbiont was observed in most investigated tick species, which has led to a “one tick species—one mutualistic symbiont” paradigm [24, 26]. However, this paradigm has recently been challenged by several studies, including the analysis of the tick *Hyalomma marginatum,* which simultaneously carries two different symbionts (*Francisella* and *Midichloria*) that combine their metabolic capabilities for fully functional biosynthetic pathways for biotin, riboflavin, and folate [27].

Despite the documented pivotal role of symbionts in many tick species, little is known about their presence in *I. frontalis*. Previous molecular screenings on a small number of specimens of *I. frontalis* (*n =* 5) revealed the presence of three bacterial genera, all already known to include endosymbionts: *Midichloria*, *Spiroplasma,* and *Rickettsia* [25]. *Midichloria* and *Rickettsia* are both members of the *Rickettsiales,* and represent the most common bacterial symbionts in the genus *Ixodes*, where *Coxiella* and *Francisella* are rarely found [28, 29]. *Midichloria mitochondrii* is found at high prevalence in *I. ricinus* and has the genomic potential to have beneficial effects on the tick, including a set of genes for biosynthesis of B vitamins [30]. Experimental observations have demonstrated that its removal has a negative impact on the host. Specifically it results in the formation of substandard larvae with limited blood feeding capacity [31, 32]. *I. scapularis* instead harbors *Rickettsia buchneri*, that possesses all the genes responsible for de novo folate biosynthesis [33]. Overall, the role of *Rickettsiales* symbionts in *Ixodes* is not fully characterized, and, based on the varying prevalences reported in epidemiological studies, appears to be potentially facultative (i.e. advantageous but not absolutely necessary for the tick), or at least more plastic than what observed in other tick genera [28].

Bacteria of the genus *Spiroplasma,* member of the *Mollicutes*, are also frequently detected in different tick genera [24, 34, 35]*.* This diverse bacterial genus, divided into three main clades, includes a variety of members that entertain relationships with multiple arthropods and plants, ranging from mutualist to pathogen. Noteworthily, *Spiroplasma* has been repeatedly reported as a reproductive manipulator of its arthropod hosts. Particularly, it can act as a male killer in the pea aphid (*Acyrthosiphon pisum*)*,* in the fruitfly (*Drosophila melanogaster*) and in the moth *Homona magnanima* [36–38], while it has been shown to cause cytoplasmic incompatibility (CI) in the parasitoid wasp *Lariophagus distinguendus* [39]. On the other hand, several strains of *Spiroplasma* are known to be defensive symbionts, protecting their arthropod hosts, including aphids and fruit flies, against parasitoid wasps or infections from nematodes and fungi [37, 40]. Some knowledge is available for the molecular mechanisms underlying these diverse effects: the Spaid gene is responsible for the male-killer phenotype in *S. poulsonii* MRSO [41], while proteins of the RIP family are responsible for the defensive phenotype in *S. poulsonii* sNeo [38].

The species *S. ixodetis* was first isolated from *I. pacificus* [42] and then found in other ticks, but also in other arthropods [35]. The effects of *Spiroplasma* bacteria in ticks are still unknown, but the documented transovarial transmission suggests its potential role as a facultative mutualist or as a manipulator of the host reproduction [43, 44].

In general, there is still limited available knowledge on the genetic and genomic diversity of bacterial symbionts within single tick species, which has been investigated until now in few species by multilocus typing studies [45–47] as well as quite recently genomic studies [48, 49].

In this study, we aim to better understand the symbiont community of *I. frontalis* and its role using molecular and genomic approaches. Specifically, we investigated the prevalence of symbionts in tick samples from two different countries, France and Italy, and we tested the relative influence of geographical provenance, host life stage, and host genetics. We also measured the symbionts’ genetic variability on marker sequences, as well as at the whole genome level on selected samples. Finally, we inferred from genome sequences the potential role of each symbiont for the tick.

## Materials and methods

### Tick sampling

**Figure 1:**
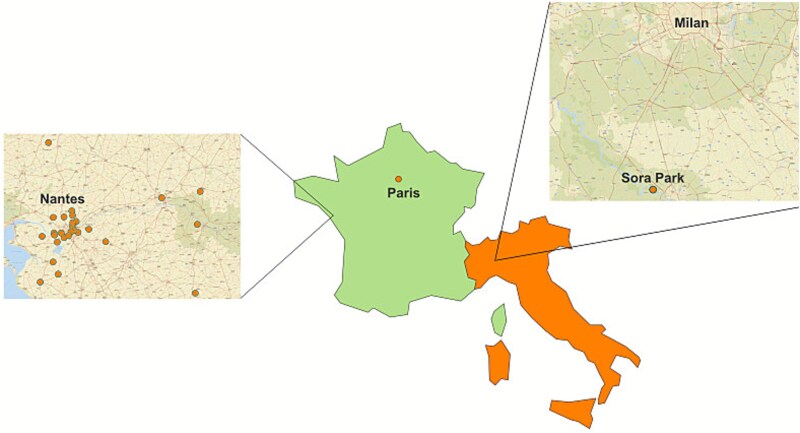
Map of the sampling locations (indicated with the orange dots) in Italy and in France, with magnifications of the areas around Nantes and Milan.

Tick sampling was conducted between November 2017 and October 2022, in 29 different sites in France and in northern Italy (Fig. 1). Ticks of all life stages were collected from the environment with the flagging technique or removed from captured birds admitted to the wildlife rescue center in the Oniris veterinary school (CVFSE). Upon collection, all ticks were placed into plastic tubes containing 70% ethanol. A total of 277 specimens were collected, comprising 57 females, four males, 53 nymphs, and 163 larvae (details of sampling location, numbers, stages and bird host species are listed in Supplementary Table 1). Ticks were identified to species using morphological keys [50, 51].

Additionally, four engorged females collected in Nantes and Chize laid eggs under laboratory conditions and the 84 newly hatched larvae were collected and placed in ethanol as above (respectively, two larvae from mother 1, 22 from mother 2, 26 from mother 3 and 34 from mother 4, the single one from Chize). During the course of this experiment, ticks (including females laying eggs before hatching, and newborn larvae before collection) were kept in closed plate containers, to avoid external contaminations.

### Molecular analyses

DNA was then extracted from each single tick specimen using the NucleoSpin Tissue kit (Macherey-Nagel, Germany), following the manufacturer’s protocol, and preserved at −20°C until usage. Extracted DNA was quantified using Qubit dsDNA Broad Range assay (Thermo Fisher, USA). The sample FG22045 was processed separately to obtain high molecular weight DNA, which was extracted using a Genomic-tip 20/G kit (Qiagen Benelux, the Netherlands). Standard PCRs were performed with the ExTaq polymerase and reagents (Takara Bio, Japan), following manufacturer’s specifications.

To characterize ticks at a genetic level and to distinguish between the two existing haplogroups, a PCR was performed to amplify a 710 bp fragment of the mitochondrial COI gene [13]. Each individual was then tested by PCR for the presence of three species of bacteria known to circulate in *I. frontalis* ticks: *Midichloria*, *Spiroplasma,* and *Rickettsia* spp. [24]. For the detection of *Midichloria*, a fragment of 16S rRNA gene was amplified with a semi-nested PCR, following the primers and protocol of Cafiso and colleagues [52] and another set of primers was newly designed targeting a fragment of 764 bp of the *fliC* gene using the annotated genome of a *Midichloria* symbiont of *I. frontalis* -GCA_030068805 [49]: Midi_flic_fw610 (3′-GTG ATC AAG TGC TTC TAA ACG-5′) and Midi_flic_rv1374 (3′-AGT AGC AAG ATC TGC AAG ATC T-5′). The amplification conditions were as follows: 95°C for 5 min, 10 cycles at 95°C for 1 min, 56°C for 1 min and 72°C for 3 min, followed by 30 cycles at 95°C for 1 min, 52°C for 1 min and 72°C for 3 min, and a final elongation of 5 min at 72°C. For the detection of *Rickettsia* spp. and *Spiroplasma* we used previously published protocols [24], respectively to amplify a fragment of 645 bp of the gene *gltA* with the primers RickF1, RickR1 and RickF2, and targeting a fragment of 822 bp of the 16S rRNA with primers SpixoF2, SpixoR1, and SpixoR2. All PCR reactions included appropriate negative and positive (i.e. tick specimens already tested to be positive for the respective PCR assay) controls.

PCR products of COI from ticks, *fliC* from *Midichloria* and 16S rRNA from *Spiroplasma* were purified with the kit NucleoSpin® Gel and PCR Clean-up kit (Macherey Nagel, Duren, Germany) following manufacturer’s instructions. Purified products were Sanger sequenced in both-directions at Eurofins Genomics (Eurofins Genomics, Ebersberg, Germany).

### Sequence analysis

Chromatograms were manually inspected and corrected with Chromas (https://technelysium.com.au/wp/chromas/). Corrected sequences were then compared to the NCBI GenBank database using BLASTN, allowing to obtain ortholog sequences from previous studies. Sequence alignments were then performed using MUSCLE [53] and polished using Gblocks with default parameters [54]. Maximum-likelihood phylogeny was inferred by IQ-TREE version 2.3.6 [55], available on Galaxy platform [56], which automatically selects the best model using Model Finder according to the Bayesian Information Criterion (BIC) [57]. Ultra-fast Bootstrap values were obtained with 1000 replications.

### Genome analyses

Three adult females were subjected to whole-genome sequencing, two of which belonging to haplogroup A and one from haplogroup B in order to investigate possible differences in the symbiont genomes between the tick lineages (specifically, samples C08 and C09 belong to haplogroup A while FG22045 to haplogroup B). For two samples (C08 and C09) Illumina short read sequencing with TruSeq DNA PCR free library was performed on NovaSeqX instrument.

For sample FG22045 the A1D ligation library was prepared from the DNA sample using the Ligation Sequencing Kit SQK-LSK110 according to the manufacturer’s instructions (Oxford Nanopore Technologies, United Kingdom). The Oxford Nanopore Technologies (ONT) library was first tested on a MinION flowcell (FLO-MIN106) and subsequently run on an R9.4.1 PromethION flowcell (FLO-PRO002) using the following settings: basecall model: high-accuracy; basecaller version: 4.0.11 (PromethION). Part of the DNA sample was used to prepare Illumina libraries using the Nextera DNA Flex Library Prep Kit (Illumina Inc. San Diego, CA, USA). The genomic paired-end (PE) libraries were sequenced with a read length of 2 × 150 nt using the Illumina NovaSeq 6000 system. The ONT sequences underwent quality assessment using Nanoplot v1.42 and Illumina sequences were assessed using MultiQC v1.14.

Short reads were assembled using SPAdes v. 3.6.0 [58], and the obtained assemblies were analysed using an in-house pipeline based on the blobology method [59]. Specifically, contigs representing the whole genome of *Spiroplasma* and *Midichloria* symbionts were selected from the preliminary assemblies based on GC composition, coverage and taxonomic annotation, and reads mapped on the selected contigs were extracted from the original reads dataset. Such thresholds, particularly for coverage, allow to discard sequences from the host and other potential co-occurring bacteria (e.g. surface microbiome), as well as to efficiently rule out possible external contaminations in the sequenced samples. From the assembly of sample C09, reads from contigs with coverage higher than 100x and with a GC content between 0.32 and 0.44 were extracted to obtain the *Midichloria* draft genome, while reads from contigs with a GC content below 0.30 were extracted to obtain the *Spiroplasma* genome. From the assembly of sample C08, contigs with a GC content lower than 0.30 were selected, and reads extracted to obtain the draft genome of *Spiroplasma*. From the three datasets, contigs annotated as eukaryotic were discarded. The new refined reads sets were then assembled in order to obtain more refined symbionts’ genomes. The ONT sequences were instead assembled using Flye 2.9.170 [60]. The final scaffolds generated by the pipeline were then polished using Illumina short reads and NextPolish v1.4.071 with three reruns [61]. Finally, a quality control of the refined assemblies was applied, by evaluating their completeness and presence of potential contaminants in comparison with reference assemblies with BUSCO v. 5.8 [62]. All the obtained genomes were annotated using PROKKA v. 1.10 [63].

Genomes belonging to representatives of genera *Spiroplasma* and *Midicholoria* were downloaded from online databases (Supplementary Table 2) to perform comparisons with the newly assembled genomes, in terms of Average Nucleotide Identity (ANI) and presence/absence of specific genes. ANI values among selected genomes were calculated using OrthoANI [64]. The pattern of presence/absence of a set of genes of interest was assessed using local BLASTP on the genome dataset, followed by manual inspection of the results.

### Statistical analyses

We used Bayesian hierarchical logistic regression to estimate how *Midichloria* and *Spiroplasma* positivity was affected by the presence of the other symbiont (e.g. an exclusion pattern), accounting for life stage, sampling location, and haplotype. We fit the models in R with the brms package implementation of Stan [65, 66], using as outcome variables the positivity to *Midichloria* and *Spiroplasma* and the default weakly informative prior distributions, with the tick stage and positivity to the other symbiont as fixed effects, and sampling location and haplotype as random effects. For each symbiont, we fitted models with random effects for only location or haplotype and for both, then used leave-one-out cross-validation to estimate the expected log pointwise predictive density (ELPD) for each model, selecting the model with the highest ELPD. For *Midichloria* the model considering both haplogroup and sampling location as random effects was selected, while for *Spiroplasma* the model with only sampling location as random effect, defining as reference level the larval stage and absence of the other symbiont. We also used a logistic regression to assess the association between the sampling nation, used as predictor, and haplogroup, as response variable, using the default weakly informative prior distributions.

## Results

### Tick lineages and symbiont prevalences

All the collected specimens were morphologically identified as *I. frontalis*, then confirmed through COI PCR and sequencing, which also determined whether a tick belonged to haplogroup A or B. The results indicate that the majority of ticks belong to haplogroup A 203/277 (73.3%), while 74/277 specimens (26.7%) belong to haplogroup B. The identity between the two haplogroups was found to be around 92%, consistent with previous results [13]. Considering the sampling sites, lineage A is always more prevalent in France, while lineage B is more prevalent in the Italian site (Table 1, see also Supplementary Table 3 and Supplementary Fig. 1 for more details). The result of the logistic regression supports a substantial difference in the haplogroup prevalence between Italy and France, with a posterior mean probability of haplogroup B of 72% in Italy (95% CI: 60%–82%) and of 18% in France (95% CI: 14%–22%).

**Table 1:** Numbers and prevalences of haplogroups A and B, divided per geographic sampling area (French administrative regions and Italian regions).

**Localities**	**Total tick number**	**Number of ticks per haplogroup (%)**	
Pays de la Loire (FR)	194	**A**	160 (82.5%)
		**B**	34 (17.5%)
Ile de France (FR)	15	**A**	12 (80%)
		**B**	3 (20%)
Lombardy (IT)	46	**A**	13 (28%)
		**B**	33 (72%)
Maine et Loire (FR)	21	**A**	17 (81%)
		**B**	4 (19%)
Bretagne (FR)	1	**A**	1 (100%)

**Figure 2:**
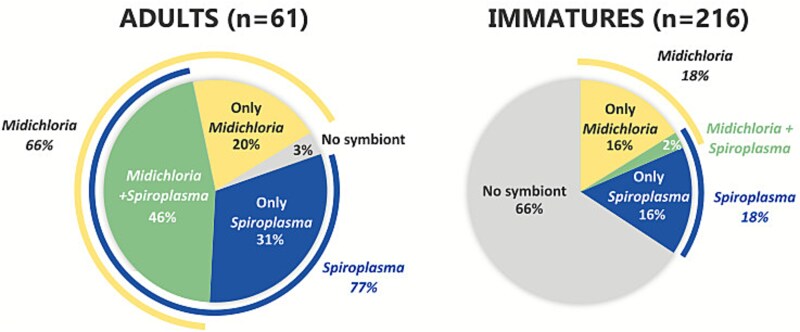
Pie charts showing the prevalence of different symbionts detected in ticks, adults (*n =* 61) and immatures (*n =* 216) are shown separately.

Molecular analysis of symbiont presence shows complete absence of *Rickettsia*, while prevalence for *Midichloria* and *Spiroplasma* varies among life stages, with a greater number of negatives among immatures than in adults (no symbionts detected in 66% of immatures, compared to 3% adults). Among adults, 66% (40/61) of samples resulted positive for *Midichloria*, 77% (47/61) for *Spiroplasma*. Of these, 46% (28/61) of adults carry both symbionts (Fig. 2, see also Supplementary Table 4).

Additionally, single larvae (*n =* 84) from four mothers were subjected to the same screening protocols, hinting for a quite efficient vertical transmission (all larvae from two mothers were positive to *Midichloria*, all larvae from a third mother were positive to *Spiroplasma*, all larvae of the fourth mother were negative to both symbionts). However, due to experimental limitations, the mother carcasses could not be tested directly.

### Statistical analyses of symbiont distribution

As expected, the Bayesian logistic regression estimated the life stages as the principal predictors for positivity: the adult life stage statistically increases the probability of presence of both symbionts (Supplementary Fig. 2). More interestingly, the presence of a symbiont statistically reduces the probability of positivity to the other one, but with limited effect, as the majority of the posterior distribution log-odds are negative, but include the zero in 90% Credibility Intervals (CIs). For what concerns the relationship between sampling location and *Midichloria* or *Spiroplasma* positivity, the random effects for location indicate variability in the positivity even if with considerable uncertainty. For instance, Lombardy has a decreased rate of positivity for *Midichloria* (baseline log odds: -0.61, CI 50%: −1.09/−0.11, CI 90%: −2.05/0.65), while an increased one for *Spiroplasma* (baseline log odds: 0.61 CI 50%: 0.21/0.99, CI 90%: −0.44/1.82). On the other hand, Angers has an increased rate of positivity for *Midichloria* (baseline log odds: 1.14; CI 50%: 0.55/1.63, CI 90%: −0.08/2.67) and a decreased one for *Spiroplasma* (baseline log odds: -0.81; CI 50%: −1.28/−0.18, CI 90%: −2.55/0.40).

The haplogroup presents a weak association with *Midichloria* positivity (haplotype A baseline log odds: 0.31, 50% CI: −0.28/1.00, 90% CI 90%: −1.86/2.19; haplotype B, baseline log odds: -1.08, 50% CI: −1.72/−0.34, 90% CI: −3.40/0.72) (See Supplementary Table 5).

### Sequence analysis of the symbionts

**Figure 3:**
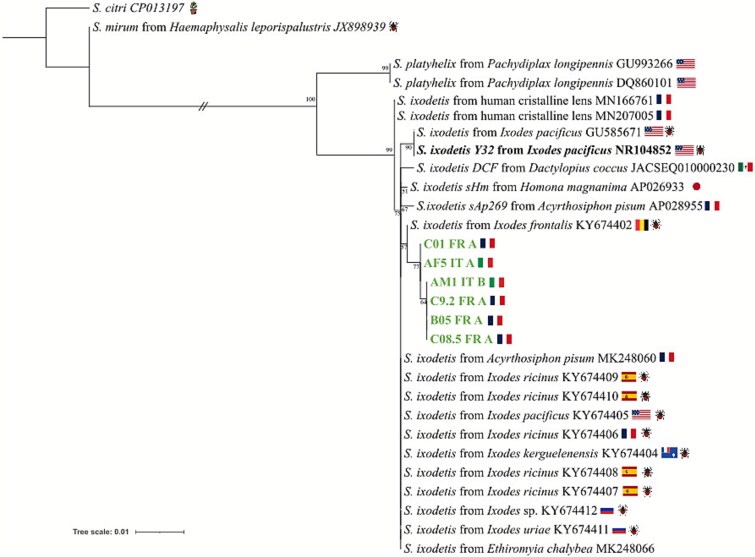
Phylogenetic tree of a fragment of the 16S rRNA gene of *Spiroplasma*. Bootstrap values under 50 are not indicated. Samples from this study are shown in green, and without accession numbers, while *S. ixodetis* Y32 is highlighted in bold, being the reference genome of *S. ixodetis* from ticks. *S. citri* (CP013197) was used as an outgroup.

Sequences of the two *Midichloria* genes (16S rRNA and *fliC*) were obtained from all positive individuals, and they all resulted identical, also showing 100% identity with the published *Midichloria* genome from *I. frontalis* [49]. *Spiroplasma* 16S rRNA gene sequences were obtained from all positive individuals and were all identical, except for three positions, two of which alternatively showed A,G, or a double peak, and the other one showing G,T, or a double peak. We attribute these small intra-sample variations to the known presence of two 16S rRNA operon copies in some *Spiroplasma* genomes [67]. BLASTN analysis revealed that all sequences belong to *S. ixodetis*. A representative subset of the obtained sequences was then used to perform a phylogenetic analysis including published *Spiroplasma* spp. sequences (Fig. 3). The tree confirmed that the novel sequences belong to *S. ixodetis*, and form a monophyletic clade with a database sequence of a symbiont of *I. frontalis* from Belgium (KY674402) with respect to *S. ixodetis* from other tick species.

### Genomics

**Figure 4:**
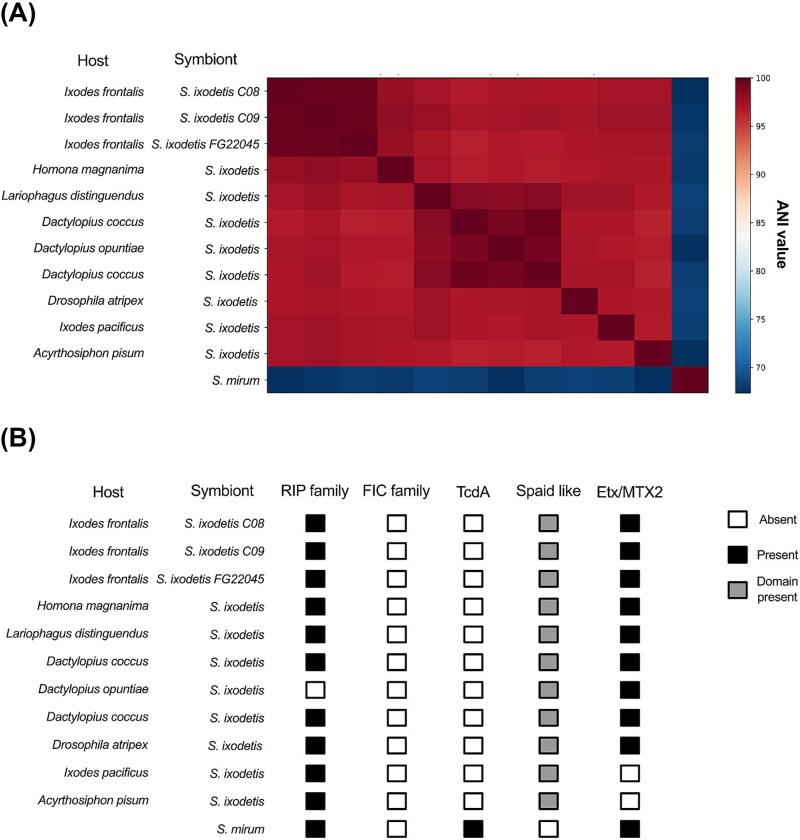
*Spiroplasma* genomic comparisons. (A) Whole genome Average Nucleotide Identity matrix among selected *Spiroplasma* representatives. Column names are in the same order as rows. (B) Gene presence/absence. Black boxes indicate the presence of orthologous genes, empty boxes absence of genes and grey indicate presence of significant BLAST hits for gene segments, possibly due to the presence of single domains (see Discussion). See supplementary Table 2 for accession numbers of the genomes.

Three adult females were subjected to shotgun sequencing to obtain symbiont genomes, respectively one sample belonging to haplogroup A with both symbionts, one from haplogroup A with only *Spiroplasma*, and one from haplogroup B with only *Spiroplasma*. The latter (FG22045) was sequenced with Nanopore long-read, allowing to retrieve a complete circular *S. ixodetis* genome. The two other WGS were performed with Illumina (samples C08 and C09), allowing to obtain one *M. mitochondrii* and two *S. ixodetis* draft genomes*.* The *Midichloria* genome from sample C09 resulted in 310 contigs for a total length of 1 208 613 bp (N50 = 20.613). *Spiroplasma* genomes from samples C08, C09 and FG22045 were respectively composed by 143 contigs (1.40 Mbp total, N50 = 19.716), 120 contigs (1.33 Mbp total, N50 = 51.072) and 1 circularized chromosome (1.43 Mbp) plus three circularized plasmids (60 946 bp, 24 849 bp and 17 242 bp respectively long). The *Midichloria* genome size is coherent with other representatives of the genus, while the three assemblies of *Spiroplasma* genomes are smaller in size with respect to other representatives of *S. ixodetis*. The quality of the four genomes in terms of completeness and contamination was confirmed by BUSCO analyses, being equivalent respectively with reference *S. ixodetis* from multiple hosts and with reference *Midichloria* from multiple tick species, including *I. frontalis* (Supplementary Table 6). We found very little differences in the three genomes of *S. ixodetis* in *I. frontalis* (ANI 99.71%), regardless of the host haplogroup (A or B) and, overall, a quite high nucleotide identity among representatives of *S. ixodetis* from various hosts (lowest ANI value 96.33%) (Fig. 4A).

**Figure 5:**
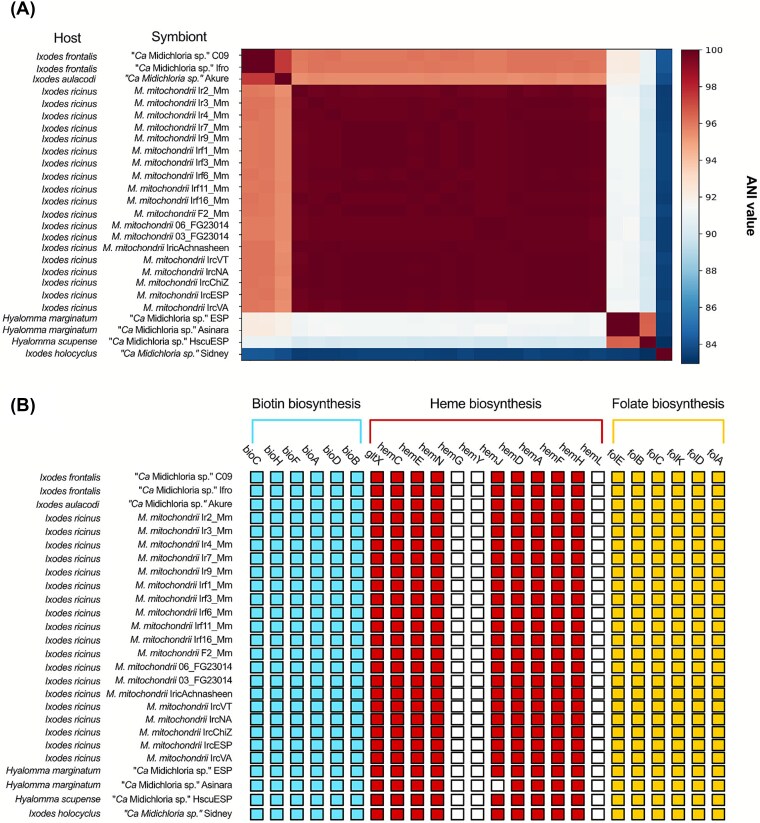
*Midichloria* genomic comparisons. (A) Whole genome Average Nucleotide Identity matrix among selected *Midichloria* representatives. (B) Gene presence/absence. Colored boxes indicate the presence of orthologous genes, empty boxes absence of the genes. See Supplementary Table 2 for accession numbers of the genomes.

The novel *Midichloria* genome, from sample C09, showed an identity of 99.99% with the previously published genome of *M. mitochondrii* from another *I. frontalis* [49] but the overall identity with the *Midichloria* genomes obtained from different tick species was relatively lower (96% between *Ixodes*, with the exception of *I. holocyclus,* 84%, and 91% with *Hyalomma* spp.) (Fig. 5A).

For gene content analyses, we focused on selected genes of interest in the genomes of the two symbionts based on previous studies [30, 68]. Genes of two key B vitamin pathways (biotin and folate) involved in nutritional symbiosis were retrieved from all *Midichloria* genomes (Fig. 5B), while they were absent in *S. ixodetis*, with the exceptions of *folD* and *folA,* involved in folate biosynthesis, and of *gltX*, involved in heme biosynthesis (which also has a housekeeping function, being a glutamate-tRNA ligase).

Conversely, several genes previously associated with host interaction and vertical transmission in *Spiroplasma* [68] were retrieved in the *S. ixodetis* genomes, specifically in the circularized chromosome for the complete genome (Fig. 4B). These genes belong to the Ribosome-inactivating proteins (RIPs) family, the pore-forming toxin Etx/MTX2 genes, and the Spaid-like gene family, although for the latter we found hits aligning only for part of the sequence (see Discussion below). No genes for FIC or TcdA toxins were found in the analyzed genomes. In various arthropod endosymbionts, including different species of *Spiroplasma*, FIC family toxins were linked to the transfer of AMP moieties to host target proteins, thereby disrupting their activity [68]. On the other hand, TcdA is a toxin involved in disrupting host cell signaling pathways [68].

## Discussion

In this study, we used a molecular approach to investigate the presence of bacterial endosymbionts in the ornithophilic hard tick *I. frontalis* and, with the use of genomic analysis, we explored the possible roles of these bacteria in tick physiology.

### Two symbionts are common, but not at fixation, in *I. frontalis*

Our molecular investigations show that both symbionts (*M. mitochondrii* and *S. ixodetis*) are present at lower prevalence in larvae and nymphs compared to adults in all the examined sites, as the overall prevalence of at least one symbiont in adults is 97%, compared to 34% in immatures (Fig. 2). This may be attributed to a generally lower amount of total DNA, due to the smaller size of immatures, making the ratio of symbiont/tick DNA insufficient for molecular detection, also considering that all immatures were nonengorged. This hypothesis is corroborated by numerous previous studies that show that the load of endosymbionts is generally low in immatures in many arthropod symbioses, even more so after periods of starvation [69]. In ticks, such levels tend to increase after blood meal and in adults [25, 46, 70, 71]. In this regard, we must consider that the presence of endosymbionts often involves some cost for the host (e.g. energetic), which for mutualists is compensated by positive effects by the bacteria, that can be variable, also depending on conditions and life stages. Consequently, it may be beneficial to the host and not necessarily disadvantageous for the symbiont to reduce the symbiont load to undetectable levels during stages in which endosymbionts are not strictly required [72, 73]. This could suggest that the two symbionts may fulfil a role in adults and/or in association with the blood meal. The higher prevalence found in newly hatched larvae in the laboratory, compared that of wild immatures, could be explained expanding this hypothesis. In this scenario, newly hatched larvae would be all positive for symbionts present in the mother, and then postmoulting they would undergo a decline in symbiont levels, as seen for *Midichloria* in *I. ricinus* [25]. Indeed, in the case of nutritional symbioses involving *Coxiella*, it has been shown that starvation can have a strong impact on symbiont density: the longer the starvation period, the lower the *Coxiella* density [69].

In adults, we detect a high prevalence of both symbionts in all the investigated populations, but considering each of the two separately, prevalence is far from 100%. Regarding *Midichloria*, the observed prevalence of 70% in females is consistent with a previous study on *I. frontalis* based on a limited number (*n =* 10) of individuals for this species [52]. Interestingly, this data differs from what reported in most other species of ixodid ticks, where this endosymbiont is either close to fixation in females in *I. ricinus*, *I. holocyclus, I. aulacodi,* and *H. marginatum* [24, 52, 74, 75] or scarcely present, with prevalences below 10% (in *H. impeltatum*, *H. dromedari, H. aegyptium,* and *Amblyomma americanum* [76–78]. Whether this indicates a distinct role in *I. frontalis* or is a hint of an ongoing process of fixation remains to be determined.

The prevalence of *S. ixodetis* in *I. frontalis* females is around 75%, higher than what reported for most tick species, such as *I. ricinus, I. arboricola, I. persulcatus, H. flava, Dermacentor nitens,* and *A. sculptum*, where the prevalence is generally low (below 20%) [24, 44, 79–82]. An even higher prevalence was reported in *I. ovatus*, where *Spiroplasma* is almost at fixation [81, 83]. The high observed prevalence suggests a potential positive contribution to the fitness of *I. frontalis* or a manipulation of host reproduction.

Statistical analyses revealed a slightly negative association between the presence of the two symbionts. This could indicate that their role is not synergistic, contrary to what is observed for the dual symbiosis in *H. marginatum*, where *M. mitochondrii* and *Francisella* were found to co-vary [27]. Multiple hypotheses could explain this negative association. These include direct active competition for host resources among symbionts, possibly mediated by signaling molecules [84, 85]. An alternative and not mutually exclusive explanation is the presence of an additive cost of the symbionts for the host. Indeed, an increase in total bacterial density in arthropods with multiple symbionts is often linked with higher cost of infection for the host [86]. Under certain circumstances, this elevated cost may impact significantly the host fitness, in turn driving selection against multiple infections. On the other hand, we identified only weak links between the presence of each symbiont and the two different *I. frontalis* mitochondrial haplogroups or the geographical locations, indicating that the presence of both bacteria is widespread, and could be evolutionary stable in this tick species.

### *Midichloria* and *Spiroplasma* show little genetic variation in *I. frontalis*

The stability of the two endosymbionts is also supported by molecular and genomic data, which taken together suggest a low intraspecific genetic variation. Specifically, we noticed a very high nucleotide identity among available genomes both within *M. mitochondrii* and *S. ixodetis*, as well as virtually absent differences at the level of single gene sequences on the whole dataset for each symbiont, regardless of the mitochondrial haplogroup or geographic origin of the tick host.. Further in-depth analyses on larger and more representative genomic datasets will be necessary in the future to confirm these findings.

In *Midichloria* the high ANI values between bacteria found in *I. frontalis* individuals are comparable to those of the stable *M. mitochondrii* symbiosis in *I. ricinus* (Fig. 5A). Conversely, the genetic variability between *Midichloria* genomes from different tick species is higher, suggesting that these genomes could represent different bacterial species. Considering the 95% ANI threshold for delimiting bacterial species [87], the *Midichloria* genus could thus be divided into three species, one including the symbionts of European *Ixodes*, one for the symbionts of *Hyalomma* spp., and a third one including the symbiont of the Australian species *I. holocyclus* [45]. On the other hand, in *S. ixodetis* we observe a low degree of genomic variability even in bacteria derived from phylogenetically unrelated hosts such as ticks and scale insects. They all exhibited ANI values above 95% (Fig. 4A), confirming with genomic data their status as a single species, and suggesting recent horizontal transfers of this symbiont.

### Genome content of the two symbionts reveals clues of mechanisms involved in bacterium-host interactions

The analysis of an additional *M. mitochondrii* genome from *I. frontalis* confirms the presence of a conserved set of genes involved in the B vitamin pathways (Fig. 5B), strengthening the hypothesis of a role as nutritional symbiont [28], as initially observed in *I. ricinus* [88] and then in all other *Midichloria*-Ixodidae associations [27, 49].Conversely, the gene repertoires of *S. ixodetis* suggest very limited nutritional capacities. However, all the analyzed genomes carry several genes that have been previously indicated as involved in bacterium-host interactions, and hypothesized to be important in vertically-transmitted symbiotic *Spiroplasma* [40]. Specifically, these include genes of the RIP family, a Spaid-like toxin, and ETX/MTX2 toxins (Fig. 4B).

RIP proteins are known to cause a defensive phenotype in other species of *Spiroplasma*, protecting the hosts from parasitic wasps and entomopathogenic nematodes [40]. ETX/MTX2 are pore-forming toxins characterized in *Clostridium perfringens*, widespread in *Spiroplasma* across the Citri and Ixodetis clades [68], but their role in host-bacterium interactions in *Spiroplasma* is currently unknown [89].

Moreover, we found genes encoding for domains exhibiting homology to the ankyrin repeats and the Ovarian Tumor (OTU) deubiquitinase domain of the Spaid toxins involved in male-killing (MK) in *S. poulsonii* [41]. Such domains are also found in the *S. ixodetis* strains that cause MK in the lepidopteran *H. magnanima* and in the pea aphid *A. pisum*. However, since the complete Spaid genes were not found in any *S. ixodetis* genome, the potential ability of the detected genes in a MK phenotype is still unproven [36, 37]. While our genomic data is not conclusive in this sense, all the few analyzed males carry *S. ixodetis*, pointing against a MK trait. However, also considering that *Spiroplasma* causing CI has been observed in the ectoparasitoid *L. distinguendus* [39], we should account for the possibility of reproductive manipulation effects caused by *S. ixodetis* in *I. frontalis*. Ongoing ecological analyses do not indicate the presence of a sex bias due to MK in wild populations of *I. frontalis* (Plantard, personal observation), but further studies are needed to establish whether *Spiroplasma* causes any reproductive manipulation also in ticks.

## Conclusions

To summarize, we determined that *M. mitochondrii* and *S. ixodetis* symbionts are widespread but not always present in *I. frontalis* adults and that, based on the subset of sequenced samples, they are genomically uniform and vertically transmitted. The genome of *M. mitochondrii* contains traits indicative of nutritional mutualism, while for *S. ixodetis* the situation is less clear. RIP genes suggest protective mutualism, while other genes associated with vertically transmitted strains could be involved in reproductive manipulation. Such a scenario suggests that the interaction between *I. frontalis* and its endosymbionts represent a potentially novel triad, a further singularity in the genus *Ixodes*, worth additional investigations.

## Supplementary Material

Supplementary_file_ycaf202

## Data Availability

All genomic sequence data are available under bioproject PRJNA1262375. Specifically, gene sequences for CoxI can be found under the accession numbers PV644577-PV644685, for *Spiroplasma* 16S rRNA under accession numbers PV639409-PV639450 and PV636719-PV636724, and for *Midichloria* FliC genes under the accession numbers are pending. Symbiont genomes under the accession numbers are pending.

## References

[1] Plantard O, Hoch T, Daveu R et al. Where to find questing *Ixodes frontalis* ticks? Under bamboo bushes! *Ticks Tick Borne Dis* 2021;12:101625. 10.1016/j.ttbdis.2020.101625 33383440

[2] Drehmann M, Chitimia-Dobler L, Lindau A et al. *Ixodes frontalis*: a neglected but ubiquitous tick species in Germany. *Exp Appl Acarol* 2019;78:79–91. 10.1007/s10493-019-00375-3 31093856

[3] Keve G, Sándor AD, Hornok S. Hard ticks (Acari: Ixodidae) associated with birds in Europe: review of literature data. *Front Vet Sci* 2022;9:928756. 36090176 10.3389/fvets.2022.928756 PMC9453168

[4] Cull B, Pietzsch ME, Hansford KM et al. Surveillance of British ticks: An overview of species records, host associations, and new records of *Ixodes ricinus* distribution. *Ticks Tick Borne Dis* 2018;9:605–14. 10.1016/j.ttbdis.2018.01.011 29426591

[5] Kar S, Yılmazer N, Akyıldız G et al. The human infesting ticks in the city of Istanbul and its vicinity with reference to a new species for Turkey. *Syst Appl Acarol* 2017;22:2245.

[6] Gilot B, Beaucournu JC, Chastel C. Collecting with the flagging method and fixing on man of *Ixodes* (Trichotoixodes) *frontalis* (panzer, 1795). *Parasite* 1997;4:197–9. 10.1051/parasite/1997042197 9296062

[7] Monks D, Fisher M, Forbes NA. *Ixodes frontalis* and avian tick-related syndrome in the United Kingdom. *J Small Anim Pract* 2006;47:451–5. 10.1111/j.1748-5827.2006.00031.x 16911113

[8] Movila A, Alekseev AN, Dubinina HV et al. Detection of tick-borne pathogens in ticks from migratory birds in the Baltic region of Russia. *Med Vet Entomol* 2013;27:113–7. 10.1111/j.1365-2915.2012.01037.x 22924442

[9] Jahfari S, Coipan EC, Fonville M et al. Circulation of four *Anaplasma phagocytophilum* ecotypes in Europe. *Parasit Vectors* 2014;7:365. 25127547 10.1186/1756-3305-7-365 PMC4153903

[10] Remesar S, Matute R, Díaz P et al. Tick-borne pathogens in ticks from urban and suburban areas of north-western Spain: importance of *Ixodes frontalis* harbouring zoonotic pathogens. *Med Vet Entomol* 2023;37:499–510. 10.1111/mve.12648 36896712

[11] Heylen D, Fonville M, Docters van Leeuwen A et al. Pathogen communities of songbird-derived ticks in Europe’s low countries. *Parasit Vectors* 2017;10:497. 29047399 10.1186/s13071-017-2423-y PMC5648423

[12] Heylen D, Sprong H, van Oers K et al. Are the specialized bird ticks, *Ixodes arboricola* and *I. Frontalis*, competent vectors for *borrelia burgdorferi* sensu lato? *Environ Microbiol* 2014;16:1081–9. 10.1111/1462-2920.12332 24237635

[13] Hornok S, Flaisz B, Takács N et al. Bird ticks in Hungary reflect western, southern, eastern flyway connections and two genetic lineages of *Ixodes frontalis* and *Haemaphysalis concinna*. *Parasit Vectors* 2016;9:101. 26912331 10.1186/s13071-016-1365-0 PMC4765043

[14] Charrier NP, Hermouet A, Hervet C et al. A transcriptome-based phylogenetic study of hard ticks (Ixodidae). *Sci Rep* 2019;9:12923. 31501478 10.1038/s41598-019-49641-9 PMC6733903

[15] Reynolds C, Kontschán J, Takács N et al. Shift in the seasonality of ixodid ticks after a warm winter in an urban habitat with notes on morphotypes of *Ixodes ricinus* and data in support of cryptic species within *Ixodes frontalis*. *Exp Appl Acarol* 2022;88:127–38. 10.1007/s10493-022-00756-1 36282440 PMC9663398

[16] Hornok S, Cutajar B, Takács N et al. On the way between Africa and Europe: molecular taxonomy of ticks collected from birds in Malta. *Ticks Tick Borne Dis* 2022;13:102001. 10.1016/j.ttbdis.2022.102001 35863119

[17] Duron O, Gottlieb Y. Convergence of nutritional symbioses in obligate blood feeders. *Trends Parasitol* 2020;36:816–25. 10.1016/j.pt.2020.07.007 32811753

[18] Bonnet SI, Binetruy F, Hernández-Jarguín AM et al. The tick microbiome: why non-pathogenic microorganisms matter in tick biology and pathogen transmission. *Front Cell Infect Microbiol* 2017;7:236. 28642842 10.3389/fcimb.2017.00236 PMC5462901

[19] Ben-Yosef M, Rot A, Mahagna M et al. *Coxiella*-like endosymbiont of *Rhipicephalus sanguineus* is required for physiological processes during ontogeny. *Front Microbiol* 2020;11:493. 32390951 10.3389/fmicb.2020.00493 PMC7188774

[20] Zhong Z, Zhong T, Peng Y et al. Symbiont-regulated serotonin biosynthesis modulates tick feeding activity. *Cell Host Microbe* 2021;29:1545–1557.e4. 10.1016/j.chom.2021.08.011 34525331

[21] Cibichakravarthy B, Shaked N, Kapri E et al. Endosymbiont-derived metabolites are essential for tick host reproductive fitness. *mSphere* 2024;9:e0069323. 38953331 10.1128/msphere.00693-23 PMC11288044

[22] Guizzo MG, Parizi LF, Nunes RD et al. A *Coxiella* mutualist symbiont is essential to the development of *Rhipicephalus microplus*. *Sci Rep* 2017;7:17554. 29242567 10.1038/s41598-017-17309-x PMC5730597

[23] Duron O, Morel O, Noël V et al. Tick-bacteria mutualism depends on B vitamin synthesis pathways. *Curr Biol* 2018;28:1896–1902.e5. 10.1016/j.cub.2018.04.038 29861133

[24] Duron O, Binetruy F, Noël V et al. Evolutionary changes in symbiont community structure in ticks. *Mol Ecol* 2017;26:2905–21. 10.1111/mec.14094 28281305

[25] Sassera D, Lo N, Bouman EAP et al. ‘Candidatus Midichloria’ endosymbionts bloom after the blood meal of the host, the hard tick *Ixodes ricinus*. *Appl Environ Microbiol* 2008;74:6138–40. 10.1128/AEM.00248-08 18689508 PMC2565945

[26] Binetruy F, Buysse M, Lejarre Q et al. Microbial community structure reveals instability of nutritional symbiosis during the evolutionary radiation of *Amblyomma* ticks. *Mol Ecol* 2020;29:1016–29. 10.1111/mec.15373 32034827

[27] Buysse M, Floriano AM, Gottlieb Y et al. A dual endosymbiosis supports nutritional adaptation to hematophagy in the invasive tick. *elife* 2021;10:e72747. 10.7554/eLife.72747 PMC8709577 34951405

[28] Duron O . Nutritional symbiosis in ticks: singularities of the genus *Ixodes*. *Trends Parasitol* 2024;40:696–706. 10.1016/j.pt.2024.06.006 38942646

[29] Narasimhan S, Swei A, Abouneameh S et al. Grappling with the tick microbiome. *Trends Parasitol* 2021;37:722–33. 10.1016/j.pt.2021.04.004 33962878 PMC8282638

[30] Olivieri E, Epis S, Castelli M et al. Tissue tropism and metabolic pathways of *Midichloria mitochondrii* suggest tissue-specific functions in the symbiosis with *Ixodes ricinus*. *Ticks Tick Borne Dis* 2019;10:1070–7. 10.1016/j.ttbdis.2019.05.019 31176662

[31] Guizzo MG, Hatalová T, Frantová H et al. *Ixodes ricinus* ticks have a functional association with *Midichloria mitochondrii*. *Front Cell Infect Microbiol* 2022;12:1081666. 10.3389/fcimb.2022.1081666 36699720 PMC9868949

[32] Militzer N, Pinecki Socias S, Nijhof AM. Changes in the microbiome associated with artificial tick feeding. *Front Microbiol* 2022;13:1050063. 10.3389/fmicb.2022.1050063 36704557 PMC9871825

[33] Hunter DJ, Torkelson JL, Bodnar J et al. The *rickettsia* endosymbiont of *Ixodes pacificus* contains all the genes of *de novo* folate biosynthesis. *PLoS One* 2015;10:e0144552. 10.1371/journal.pone.0144552 26650541 PMC4674097

[34] Gasparich GE, Kuo C-H, Foissac X. *Spiroplasma*. In: Bergey’s Manual of Systematics of Archaea and Bacteria. United States of America: John Wiley & Sons, 2020, 1–52.

[35] Binetruy F, Bailly X, Chevillon C et al. Phylogenetics of the *Spiroplasma ixodetis* endosymbiont reveals past transfers between ticks and other arthropods. *Ticks Tick Borne Dis* 2019;10:575–84. 10.1016/j.ttbdis.2019.02.001 30744948

[36] Arai H, Inoue MN, Kageyama D. Male-killing mechanisms vary between species. *Front Microbiol* 2022;13:1075199. 36519169 10.3389/fmicb.2022.1075199 PMC9742256

[37] Arai H, Legeai F, Kageyama D et al. Genomic insights into *Spiroplasma* endosymbionts that induce male-killing and protective phenotypes in the pea aphid. *FEMS Microbiol Lett* 2024;9:371. 10.1093/femsle/fnae027 38632047

[38] Ballinger MJ, Perlman SJ. Generality of toxins in defensive symbiosis: ribosome-inactivating proteins and defense against parasitic wasps in *drosophila*. *PLoS Pathog* 2017;13:e1006431. 10.1371/journal.ppat.1006431 28683136 PMC5500355

[39] Pollmann M, Moore LD, Krimmer E et al. Highly transmissible cytoplasmic incompatibility by the extracellular insect symbiont. *iScience* 2022;25:104335. 10.1016/j.isci.2022.104335 35602967 PMC9118660

[40] Ballinger MJ, Perlman SJ. The defensive *Spiroplasma*. *Curr Opin Insect Sci* 2019;32:36–41. 10.1016/j.cois.2018.10.004 31113629

[41] Harumoto T, Lemaitre B. Male-killing toxin in a bacterial symbiont of *drosophila*. *Nature* 2018;557:252–5. 10.1038/s41586-018-0086-2 29720654 PMC5969570

[42] Tully JG, Rose DL, Yunker CE et al. *Spiroplasma ixodetis* sp. nov., a new species from *Ixodes pacificus* ticks collected in Oregon. *Int J Syst Bacteriol* 1995;45:23–8. 10.1099/00207713-45-1-23 7857803

[43] Ogata S, Umemiya-Shirafuji R, Kusakisako K et al. Investigation of vertical and horizontal transmission of *Spiroplasma* in ticks under laboratory conditions. *Sci Rep* 2023;13:13265. 37582809 10.1038/s41598-023-39128-z PMC10427632

[44] Van Oosten AR, Duron O, Heylen DJA. Sex ratios of the tick *Ixodes arboricola* are strongly female-biased, but there are no indications of sex-distorting bacteria. *Ticks Tick Borne Dis* 2018;9:307–13. 10.1016/j.ttbdis.2017.11.004 29150322

[45] Buysse M, Duron O. Multi-locus phylogenetics of the *Midichloria* endosymbionts reveals variable specificity of association with ticks. *Parasitology* 2018;145:1969–78. 10.1017/S0031182018000793 29779502

[46] Al-Khafaji AM, Clegg SR, Pinder AC et al. Multi-locus sequence typing of *Ixodes ricinus* and its symbiont *Candidatus* Midichloria mitochondrii across Europe reveals evidence of local co-cladogenesis in Scotland. *Ticks Tick Borne Dis* 2019;10:52–62. 10.1016/j.ttbdis.2018.08.016 30197267

[47] Buysse M, Duron O. Evidence that microbes identified as tick-borne pathogens are nutritional endosymbionts. *Cell* 2021;184:2259–60. 10.1016/j.cell.2021.03.053 33930290

[48] Lesiczka PM, Azagi T, Krawczyk AI et al. Deep sequencing of 16 *Ixodes ricinus* ticks unveils insights into their interactions with endosymbionts. *mSystems* 2025;10:e0050725. 10.1128/msystems.00507-25 PMC12282096 40521888

[49] Floriano AM, Batisti Biffignandi G, Castelli M et al. The evolution of intramitochondriality in *Midichloria* bacteria. *Environ Microbiol* 2023;25:2102–17. 10.1111/1462-2920.16446 37305924

[50] Agoulon A, Hoch T, Heylen D et al. Unravelling the phenology of *Ixodes frontalis*, a common but understudied tick species in Europe. *Ticks Tick Borne Dis* 2019;10:505–12. 10.1016/j.ttbdis.2018.12.009 30612949

[51] Estrada-Peña A, Mihalca AD, Petney TN. Ticks of Europe and North Africa: A Guide to Species Identification. Switzerland: Springer, 2018.

[52] Cafiso A, Bazzocchi C, De Marco L et al. Molecular screening for *Midichloria* in hard and soft ticks reveals variable prevalence levels and bacterial loads in different tick species. *Ticks Tick Borne Dis* 2016;7:1186–92. 10.1016/j.ttbdis.2016.07.017 27521265

[53] Edgar RC . MUSCLE: multiple sequence alignment with high accuracy and high throughput. *Nucleic Acids Res* 2004;32:1792–7. 10.1093/nar/gkh340 15034147 PMC390337

[54] Castresana J . Selection of conserved blocks from multiple alignments for their use in phylogenetic analysis. *Mol Biol Evol* 2000;17:540–52. 10.1093/oxfordjournals.molbev.a026334 10742046

[55] Nguyen L-T, Schmidt HA, von Haeseler A et al. IQ-TREE: a fast and effective stochastic algorithm for estimating maximum-likelihood phylogenies. *Mol Biol Evol* 2015;32:268–74. 10.1093/molbev/msu300 25371430 PMC4271533

[56] Community G . The galaxy platform for accessible, reproducible, and collaborative data analyses: 2024 update. *Nucleic Acids Res* 2024;52:W83–94. 10.1093/nar/gkae410 38769056 PMC11223835

[57] Kalyaanamoorthy S, Minh BQ, Wong TKF et al. ModelFinder: fast model selection for accurate phylogenetic estimates. *Nat Methods* 2017;14:587–9. 10.1038/nmeth.4285 28481363 PMC5453245

[58] Bankevich A, Nurk S, Antipov D et al. SPAdes: a new genome assembly algorithm and its applications to single-cell sequencing. *J Comput Biol* 2012;19:455–77. 10.1089/cmb.2012.0021 22506599 PMC3342519

[59] Kumar S, Jones M, Koutsovoulos G et al. Blobology: exploring raw genome data for contaminants, symbionts and parasites using taxon-annotated GC-coverage plots. *Front Genet* 2013;4:237. 10.3389/fgene.2013.00237 24348509 PMC3843372

[60] Kolmogorov M, Yuan J, Lin Y et al. Assembly of long, error-prone reads using repeat graphs. *Nat Biotechnol* 2019;37:540–6. 10.1038/s41587-019-0072-8 30936562

[61] Hu J, Fan J, Sun Z et al. NextPolish: a fast and efficient genome polishing tool for long-read assembly. *Bioinformatics* 2020;36:2253–5. 10.1093/bioinformatics/btz891 31778144

[62] Manni M, Berkeley MR, Seppey M et al. BUSCO: assessing genomic data quality and beyond. *Curr Protoc* 2021;1:e323. 34936221 10.1002/cpz1.323

[63] Seemann T . Prokka: rapid prokaryotic genome annotation. *Bioinformatics* 2014;30:2068–9. 10.1093/bioinformatics/btu153 24642063

[64] Yoon S-H, Ha S-M, Lim J et al. A large-scale evaluation of algorithms to calculate average nucleotide identity. *Antonie Van Leeuwenhoek* 2017;110:1281–6. 10.1007/s10482-017-0844-4 28204908

[65] Bürkner P-C . Brms: An R package for Bayesian multilevel models using Stan. *J Stat Softw* 2017;80:1–28. 10.18637/jss.v080.i01

[66] Carpenter B, Gelman A, Hoffman MD et al. Stan: a probabilistic programming language. *J Stat Softw* 2017;76:1–32. 10.18637/jss.v076.i01 PMC9788645 36568334

[67] Vera-Ponce León A, Dominguez-Mirazo M, Bustamante-Brito R et al. Functional genomics of a *Spiroplasma* associated with the carmine cochineals *Dactylopius coccus* and *Dactylopius opuntiae*. *BMC Genomics* 2021;22:240. 33823812 10.1186/s12864-021-07540-2 PMC8025503

[68] Massey JH, Newton ILG. Diversity and function of arthropod endosymbiont toxins. *Trends Microbiol* 2022;30:185–98. 10.1016/j.tim.2021.06.008 34253453 PMC8742837

[69] An L, Bhowmick B, Liang D et al. The microbiota changes of the brown dog tick, *Rhipicephalus sanguineus* under starvation stress. *Front Physiol* 2022;13:932130. 36160860 10.3389/fphys.2022.932130 PMC9504665

[70] Liu J-N, Yu Z-J, Liu L-M et al. Identification, distribution and population dynamics of *Francisella*-like endosymbiont in *Haemaphysalis doenitzi* (Acari: Ixodidae). *Sci Rep* 2016;6:35178. 27731377 10.1038/srep35178 PMC5059625

[71] Umanzor EF, Kelly SE, Ravenscraft A et al. The facultative intracellular symbiont *Lariskella* is neutral for lifetime fitness and spreads through cytoplasmic incompatibility in the leaffooted bug, Leptoglossus zonatus. Front Microbiol 16:1595917. 10.3389/fmicb.2025.1595917 PMC12288687 40708921

[72] Vorburger C, Gouskov A. Only helpful when required: a longevity cost of harbouring defensive symbionts. *J Evol Biol* 2011;24:1611–7. 10.1111/j.1420-9101.2011.02292.x 21569156

[73] Polin S, Simon J-C, Outreman Y. An ecological cost associated with protective symbionts of aphids. *Ecol Evol* 2014;4:826–30. 10.1002/ece3.991 24683464 PMC3967907

[74] Di Lecce I, Bazzocchi C, Cecere JG et al. Patterns of *Midichloria* infection in avian-borne African ticks and their trans-Saharan migratory hosts. *Parasit Vectors* 2018;11:106. 29471857 10.1186/s13071-018-2669-z PMC5824480

[75] Beninati T, Riegler M, Vilcins I-ME et al. Absence of the symbiont *Candidatus* Midichloria mitochondrii in the mitochondria of the tick *Ixodes holocyclus*. *FEMS Microbiol Lett* 2009;299:241–7. 10.1111/j.1574-6968.2009.01757.x 19732154

[76] Selmi R, Ben Said M, Mamlouk A et al. Molecular detection and genetic characterization of the potentially pathogenic *Coxiella burnetii* and the endosymbiotic *Candidatus* Midichloria mitochondrii in ticks infesting camels (*Camelus dromedarius*) from Tunisia. *Microb Pathog* 2019;136:103655. 10.1016/j.micpath.2019.103655 31398530

[77] Barradas PF, Lima C, Cardoso L et al. Molecular evidence of *Hemolivia mauritanica*, *Ehrlichia* spp. and the endosymbiont *Candidatus* Midichloria Mitochondrii in *Hyalomma aegyptium* infesting *Testudo graeca* tortoises from Doha, Qatar. *Animals (Basel)* 2020;11:30. 10.3390/ani11010030 PMC7824506 33375268

[78] Williams-Newkirk AJ, Rowe LA, Mixson-Hayden TR et al. Presence, genetic variability, and potential significance of ‘*Candidatus* Midichloria mitochondrii’ in the lone star tick *Amblyomma americanum*. *Exp Appl Acarol* 2012;58:291–300. 10.1007/s10493-012-9582-5 22678102 PMC5730336

[79] Subramanian G, Sekeyova Z, Raoult D et al. Multiple tick-associated bacteria in *Ixodes ricinus* from Slovakia. *Ticks Tick Borne Dis* 2012;3:406–10. 10.1016/j.ttbdis.2012.10.001 23182274

[80] Beliavskaia A, Hönig V, Erhart J et al. *Spiroplasma* isolated from third-generation laboratory colony *Ixodes persulcatus* ticks. *Front Vet Sci* 2021;8:659786. 33842580 10.3389/fvets.2021.659786 PMC8032855

[81] Ogata S, Mohamed WMA, Kusakisako K et al. *Spiroplasma* infection among ixodid ticks exhibits species dependence and suggests a vertical pattern of transmission. *Microorganisms* 2021;9:333. 10.3390/microorganisms9020333 PMC7915285 33567677

[82] da Silva NX, Dias TS, Vignoli JA et al. First molecular detection of *Spiroplasma* spp. in ticks from horses in Brazil. *Ticks Tick Borne Dis* 2022;13:101896. 10.1016/j.ttbdis.2022.101896 35051893

[83] Qiu Y, Nakao R, Ohnuma A et al. Microbial population analysis of the salivary glands of ticks; a possible strategy for the surveillance of bacterial pathogens. *PLoS One* 2014;9:e103961. 10.1371/journal.pone.0103961 25089898 PMC4121176

[84] Guckes KR, Yount TA, Steingard CH et al. Quorum sensing inhibits interference competition among bacterial symbionts within a host. *Curr Biol* 2023;33:4244–4251.e4. 10.1016/j.cub.2023.08.051 37689064 PMC10592073

[85] McIlroy SE, Cunning R, Baker AC et al. Competition and succession among coral endosymbionts. *Ecol Evol* 2019;9:12767–78. 10.1002/ece3.5749 31788212 PMC6875658

[86] Ferrari J, Vavre F. Bacterial symbionts in insects or the story of communities affecting communities. *Philos Trans R Soc Lond Ser B Biol Sci* 2011;366:1389–400. 21444313 10.1098/rstb.2010.0226 PMC3081568

[87] Richter M, Rosselló-Móra R. Shifting the genomic gold standard for the prokaryotic species definition. *Proc Natl Acad Sci USA* 2009;106:19126–31. 10.1073/pnas.0906412106 19855009 PMC2776425

[88] Sassera D, Lo N, Epis S et al. Phylogenomic evidence for the presence of a flagellum and cbb(3) oxidase in the free-living mitochondrial ancestor. *Mol Biol Evol* 2011;28:3285–96. 10.1093/molbev/msr159 21690562

[89] Moore LD, Ballinger MJ. The toxins of vertically transmitted *Spiroplasma*. *Front Microbiol* 2023;14:1148263. 10.3389/fmicb.2023.1148263 37275155 PMC10232968

