# Supplementary material for: Genetic and genomic variability of Spiroplasma and Midichloria endosymbionts associated with the tick Ixodes frontalis
Source: ISME Commun. 2025 Nov 10;5(1):ycaf202. doi: 10.1093/ismeco/ycaf202 (PMC12645838; doi:10.1093/ismeco/ycaf202)
Supplement: Supplementary_file_ycaf202 [file supplementary_file_ycaf202.docx]

# Supplementary material

**Supplementary Table 1**. Details of total number of ticks sampled divided per sampling site. Developmental stages and also captured bird species are indicated, while “vegetation” indicates sampling with flagging technique.

| **Sampling site** | **Department/**  **Province** | **Total number** | **Developmental stage** | **Vegetation/ host** |
| --- | --- | --- | --- | --- |
| Angers | Maine-et-Loire (FR) | 21 | 12 nymphs +  9 larvae | Vegetation |
| Basse-Goulaine | Loire-Atlantique (FR) | 1 | 1 female | host (*Streptopelia decaocto*) |
| Beaujoire | Nantes (FR) | 23 | 23 larvae | Vegetation |
| Bouaye | Loire-Atlantique (FR) | 1 | 1 female | host (*Streptopelia decaocto*) |
| Bouguenais | Loire-Atlantique (FR) | 2 | 2 females | host (*Turdus merula*) |
| Carquefou | Loire-Atlantique (FR) | 1 | 1 female | host (*Streptopelia decaocto*) |
| Challans | Vendée (FR) | 1 | 1 female | host (*Asio otus*) |
| Parc Chantrerie | Nantes (FR) | 17 | 17 larvae | Vegetation |
| Commequiers | Vendée (FR) | 1 | 1 female | host (*Columba palumbus*) |
| Couëron | Loire-Atlantique (FR) | 4 | 4 females | 2 hosts (*Streptopelia decaocto*) |
| Parc du Grand Blottereau | Nantes (FR) | 40 | 40 larvae | Vegetation |
| Jardin des Plantes | Nantes (FR) | 20 | 20 larvae | Vegetation |
| La Herinière | Sucè sur Erdre (FR) | 14 | 14 larvae | Vegetation |
| La Marne | Loire-Atlantique (FR) | 1 | 1 female | host (*Athena noctua*) |
| Le Loroux-Bottereau | Loire-Atlantique (FR) | 9 | 9 females | 9 hosts (*Passer domesticus)* |
| Mauves-sur-Loire | Loire-Atlantique (FR) | 1 | 1 female | host (*Turdus merula*) |
| Nantes | Nantes (FR) | 7 | 7 females | 3 hosts (*Phylloscopus trochilus* and  *Streptopelia decaocto*) |
| Orvault | Loire-Atlantique (FR) | 5 | 5 nymphs | Vegetation |
| Parco della Sora | Pavia (IT) | 46 | 20 larvae +  21 nymphs +  4 males +  1 female | Vegetation |
| Rezé | Loire-Atlantique (FR) | 20 | 20 larvae | Vegetation |
| Riaillé | Loire-Atlantique (FR) | 1 | 1 female | host (*Buteo buteo*) |
| Rouans | Loire-Atlantique (FR) | 1 | 1 female | host (*Asio otus*) |
| Saint Herblain | Loire-Atlantique (FR) | 6 | 6 females | host (*Streptopelia decaocto*) |
| Saint-Jean-de-Boiseau | Loire-Atlantique (FR) | 2 | 2 females | host (*Sturnus vulgaris*) |
| Saint-Malo-de-Phily | Brittany (FR) | 1 | 1 female | host (*Buteo buteo*) |
| Sucé-sur-Erdre | Loire-Atlantique (FR) | 12 | 12 females | host (*Sturnus vulgaris*) |
| Tillières | Maine-et-Loire (FR) | 1 | 1 female | host (*Tyto alba*) |
| Treillières | Loire-Atlantique (FR) | 2 | 2 females | 2 host (*Passer domesticus*  and *Turdus philomelos*) |
| Vigneux-de-Bretagne | Loire-Atlantique (FR) | 1 | 1 female | host (*Columba palumbus*) |
| Bois de Vincennes | Paris (FR) | 15 | 15 nymphs | Vegetation |

**Supplementary Table 2.** List of genomes used for ANI and gene presence/absence comparison

| **Species name** | **Strain** | **Host name** | **Accession** |
| --- | --- | --- | --- |
| *Midichloria mitochondrii* | IrcVa | *Ixodes ricinus* | GCA_000219355 |
| *Midichloria mitochondrii* | IrcNa | *Ixodes ricinus* | GCA_030068765 |
| *Midichloria mitochondrii* | IrcESP | *Ixodes ricinus* | GCA_030068875 |
| *Midichloria mitochondrii* | IrcAchnasheen | *Ixodes ricinus* | GCA_030073385 |
| *Midichloria mitochondrii* | IrcVT | *Ixodes ricinus* | GCA_030068755 |
| *Midichloria mitochondrii* | IrcChiz | *Ixodes ricinus* | GCA_030068745 |
| “*Ca.* Midichloria sp.” | Ifro | *Ixodes frontalis* | GCA_030068805 |
| “*Ca.* Midichloria sp.” | Akure | *Ixodes aulacodi* | GCA_030068825 |
| “*Ca.* Midichloria sp.” | Asinara | *Hyalomma marginatum* | GCA_910592865 |
| “*Ca.* Midichloria sp.” | ESP | *Hyalomma marginatum* | GCA_910592695 |
| “*Ca.* Midichloria sp.” | HscuESP | *Hyalomma scupense* | GCA_030073355 |
| “*Ca.* Midichloria sp.” | Sidney | *Ixodes holocyclus* | GCA_030060845 |
| *Spiroplasma ixodetis* | Y32 | *Ixodes pacificus* | NZ_CP127039 |
| *Spiroplasma ixodetis* | sAp269 | *Acyrthosiphon pisum* | GCA_040369795 |
| *Spiroplasma ixodetis* | DO | *Dactylopius opuntiae* | GCA_017847675 |
| *Spiroplasma ixodetis* | DCM | *Dactylopius coccus* | GCA_017847655 |
| *Spiroplasma ixodetis* | DCF | *Dactylopius coccus* | GCA_017847635 |
| *Spiroplasma ixodetis* | SHM | *Homona magnanima* | GCA_027923845 |
| *Spiroplasma ixodetis* | STU | *Lariophagus distinguendus* | GCF_023846195 |
| *Spiroplasma ixodetis* | sAtri | *Drosophila atripex* | GCA_028622045 |
| *Spiroplasma mirum* | ATCC 29335 | - | GCA_000517365 |

**Supplementary Table 3**. Numbers and prevalences of haplogroups A and B, divided per geographic sampling area.

| **Sites** | **Total number of ticks** | **Haplogroup numbers (%)** | |
| --- | --- | --- | --- |
| Angers | 21 | **A** | 17 (81%) |
|  |  | **B** | 4 (19%) |
| Beaujoire | 23 | **A** | 19 (83%) |
|  |  | **B** | 4 (17%) |
| Chantrerie | 17 | **A** | 16 (94%) |
|  |  | **B** | 1 (6%) |
| Grand Blottereau | 40 | **A** | 36 (90%) |
|  |  | **B** | 4 (10%) |
| Jardin des Plantes | 20 | **A** | 17 (85%) |
|  |  | **B** | 3 (15%) |
| La Hérinière | 14 | **A** | 14 (100%) |
|  |  | **B** | 0 |
| Le Loroux Bottereaux | 9 | **A** | 7 (78%) |
|  |  | **B** | 2 (22%) |
| Nantes | 7 | **A** | 7 (100%) |
|  |  | **B** | 0 |
| Orvault Provotiere | 5 | **A** | 5 (100%) |
|  |  | **B** | 0 |
| Parco della Sora | 46 | **A** | 13 (28%) |
|  |  | **B** | 33 (72%) |
| Rezè | 20 | **A** | 5 (25%) |
|  |  | **B** | 15 (75%) |
| Saint Herblain | 6 | **A** | 6 (100%) |
|  |  | **B** | 0 |
| Sucé sur Erdre | 12 | **A** | 10 (83%) |
|  |  | **B** | 2 (17%) |
| Vincennes | 15 | **A** | 12 (80%) |
|  |  | **B** | 3 (20%) |


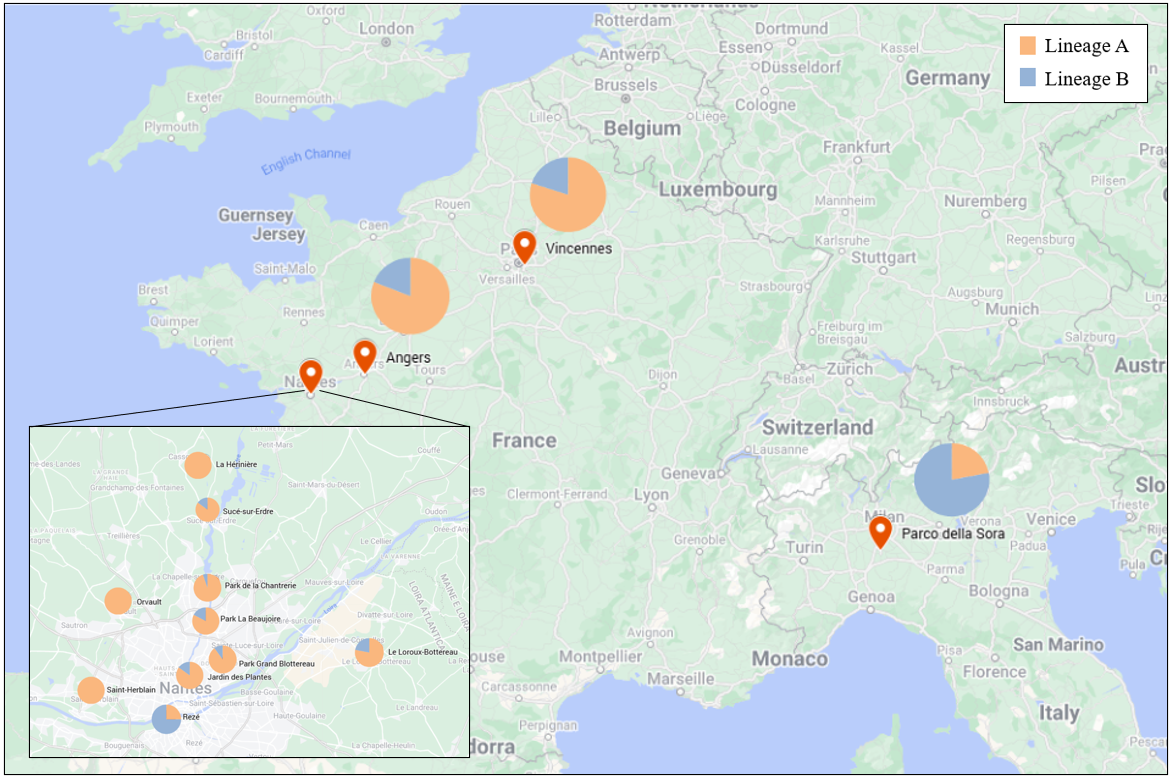


**Supplementary Figure 1**. Maps with pie charts showing the distribution of the two haplotypic lineages of *I. frontalis* in the investigated sites, considering only the sites with ≥5 specimens.


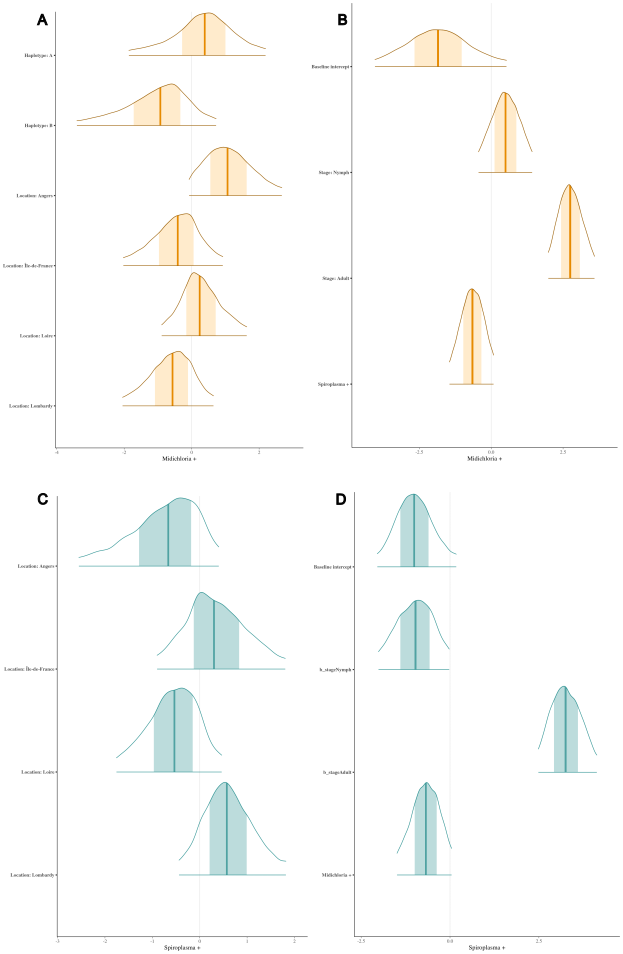


**Supplementary Figure 2:** Posterior distributions of the effects on *Midichloria*/*Spiroplasma* positivity. The x-axis contains the regression coefficients on the log-odds scale, while the y-axis the posterior density. The curves indicate the 90 % Credible Intervals (CI), the shaded areas the 50 % CI, and the vertical line the median estimate. **A**: Random effects of location and haplotype on *Midichloria* positivity; **B**: Effect of life stage and *Spiroplasma* positivity on *Midichloria* positivity; **C**: Random effects of location on *Spiroplasma* positivity; **D**: Effect of life stage and *Midichloria* positivity on *Spiroplasma* positivity.

**Supplementary Table 4.** Numbers and prevalences of *Midichloria* and *Spiroplasma* in all specimens collected in this study, divided by developmental stages.

|  | ***Midichloria* only** | ***Spiroplasma* only** | ***Spiroplasma + Midichloria*** | **No symbiont detected** |
| --- | --- | --- | --- | --- |
| **Females** | 21% (12/57) | 26.5% (15/57) | 49% (28/57) | 3.5% (2/57) |
| **Males** | 0% (0/4) | 100% (4/4) | 0% (0/4) | 0% (0/4) |
| **Adults** | 20% (12/61) | 31% (19/61) | 46% (28/61) | 3% (2/61) |
| **Nymphs** | 17% (9/53) | 11.5% (6/53) | 3.5% (2/53) | 68% (36/53) |
| **Larvae** | 16% (26/163) | 17% (28/163) | 2% (3/163) | 65% (106/163) |
| **Immatures** | 16% (35/216) | 16% (34/216) | 2% (5/216) | 66% (142/216) |

**Supplementary Table 5.** Posterior log-odds for *Midichloria* or *Spiroplasma* positivity estimated in the Bayesian logistic regression, with estimated mean, error and the 50% and 90% Credibility Intervals (CI).

| **Parameter** | **Estimate** | **Est.Error** | **CI 50%** | **CI 90%** |
| --- | --- | --- | --- | --- |
| *Midichloria* + |  | | | |
| b_Intercept | -1.83 | 1.4 | -2.67/-1.03 | -4.04/0.53 |
| b_stageNymph | 0.5 | 0.57 | 0.12/0.88 | -0.44/1.42 |
| b_stageAdult | 2.77 | 0.49 | 2.43/3.08 | 1.99/3.59 |
| b_Spiroplasma | -0.66 | 0.47 | -0.97/-0.34 | -1.45/0.08 |
| sd_Haplotype__Intercept | 1.89 | 1.43 | 0.95/2.39 | 0.49/4.53 |
| sd_location__Intercept | 1.36 | 0.88 | 0.78/1.71 | 0.37/3 |
| Intercept | -1.33 | 1.38 | -2.14/-0.55 | -3.49/1.01 |
| r_Haplotype[A,Intercept] | 0.31 | 1.26 | -0.28/1 | -1.86/2.19 |
| r_Haplotype[B,Intercept] | -1.08 | 1.29 | -1.72/-0.34 | -3.4/0.72 |
| r_location[Lombardy,Intercept] | -0.62 | 0.86 | -1.09/-0.11 | -2.05/0.65 |
| r_location[Ile,Intercept] | -0.47 | 0.93 | -0.97/0.06 | -2.02/0.92 |
| r_location[Angers,Intercept] | 1.14 | 0.88 | 0.55/1.63 | -0.08/2.67 |
| r_location[Loire,Intercept] | 0.29 | 0.8 | -0.16/0.72 | -0.89/1.63 |
| *Spiroplasma +* |  | | | |
| b_Intercept | -0.98 | 0.69 | -1.4/-0.6 | -2.05/0.18 |
| b_stageNymph | -0.99 | 0.61 | -1.4/-0.57 | -2.01/-0.02 |
| b_stageAdult | 3.27 | 0.5 | 2.92/3.6 | 2.49/4.13 |
| b_Midichloria | -0.7 | 0.47 | -0.99/-0.37 | -1.49/0.04 |
| sd_location__Intercept | 1.17 | 0.76 | 0.66/1.48 | 0.32/2.59 |
| Intercept | -0.65 | 0.66 | -1.02/-0.29 | -1.66/0.44 |
| r_location[Lombardy,Intercept] | 0.61 | 0.69 | 0.21/0.99 | -0.44/1.82 |
| r_location[Ile,Intercept] | 0.37 | 0.83 | -0.13/0.83 | -0.9/1.81 |
| r_location[Angers,Intercept] | -0.81 | 0.93 | -1.28/-0.18 | -2.55/0.40 |
| r_location[Loire,Intercept] | -0.58 | 0.7 | -0.97/-0.15 | -1.76/0.46 |

**Supplementary Table 6.** BUSCO statistics for all the analyzed genomes.

| Organism | Strain | Complete orthologs | Single copy orthologs | Duplicated orthologs | Fragmented orthologs | Missing orthologs |
| --- | --- | --- | --- | --- | --- | --- |
| *S. ixodetis* | FG22045 | 72.6% | 72.6% | 0.0% | 7.3% | 20.1% |
| *S. ixodetis* | C08 | 71.8% | 71.8% | 0.0% | 7.3% | 20.9% |
| *S. ixodetis* | C09 | 72.6% | 72.6% | 0.0% | 7.3% | 20.1% |
| *S. ixodetis* | Y32 | 70.2% | 70.2% | 0.0% | 8.9% | 20.9% |
| *S. ixodetis* | STU | 67.7% | 67.7% | 0.0% | 8.9% | 23.4% |
| *S. ixodetis* | sAp269 | 68.5% | 68.5% | 0.0% | 8.9% | 22.6% |
| *S. ixodetis* | sAtri | 71.8% | 71.8% | 0.0% | 8.1% | 20.1% |
| *S. ixodetis* | SHM | 66.9% | 66.9% | 0.0% | 8.9% | 24.2% |
| *S. ixodetis* | DO | 67.7% | 67.7% | 0.0% | 8.1% | 24.2% |
| *S. ixodetis* | DCM | 69.4% | 69.4% | 0.0% | 8.1% | 22.5% |
| *S. ixodetis* | DCF | 47.6% | 47.6% | 0.0% | 8.1% | 44.3% |
| *S. mirum* | ATCC 29335 | 78.2% | 78.2% | 0.0% | 3.2% | 18.6% |
| *“Ca.* Midichloria sp.” | C09 | 82.6% | 82.6% | 0.0% | 8.0% | 9.4% |
| *“Ca.* Midichloria sp.” | Ir2_Mm | 85.5% | 84.7% | 0.8% | 4.8% | 9.7% |
| *“Ca.* Midichloria sp.” | Ir3_Mm | 86.3% | 86.3% | 0.0% | 4.8% | 8.9% |
| *“Ca.* Midichloria sp.” | Ir4_Mm | 87.1% | 87.1% | 0.0% | 4.0% | 8.9% |
| *“Ca.* Midichloria sp.” | Ir7_Mm | 87.1% | 87.1% | 0.0% | 4.0% | 8.9% |
| *“Ca.* Midichloria sp.” | Ir9_Mm | 87.1% | 87.1% | 0.0% | 4.0% | 8.9% |
| *“Ca.* Midichloria sp.” | Irf11_Mm | 87.1% | 87.1% | 0.0% | 4.0% | 8.9% |
| *“Ca.* Midichloria sp.” | Irf16_Mm | 87.1% | 87.1% | 0.0% | 4.0% | 8.9% |
| *“Ca.* Midichloria sp.” | Irf1_Mm | 87.1% | 87.1% | 0.0% | 4.0% | 8.9% |
| *“Ca.* Midichloria sp.” | Irf3_Mm | 87.1% | 87.1% | 0.0% | 4.0% | 8.9% |
| *“Ca.* Midichloria sp.” | Irf6_Mm | 87.1% | 87.1% | 0.0% | 4.0% | 8.9% |
| *“Ca.* Midichloria sp.” | 03_FG23014 | 87.1% | 87.1% | 0.0% | 4.0% | 8.9% |
| *“Ca.* Midichloria sp.” | 06_FG23014 | 87.1% | 87.1% | 0.0% | 4.0% | 8.9% |
| *“Ca.* Midichloria sp.” | F2_Mm | 87.1% | 87.1% | 0.0% | 4.0% | 8.9% |
| *“Ca.* Midichloria sp.” | HscuESP | 75.8% | 75.8% | 0.0% | 9.7% | 14.5% |
| *“Ca.* Midichloria sp.” | Sidney | 82.3% | 82.3% | 0.0% | 6.5% | 11.2% |
| *M. mitochondrii* | IrcChiz | 84.7% | 84.7% | 0.0% | 5.6% | 9.7% |
| *M. mitochondrii* | IrcVT | 85.5% | 85.5% | 0.0% | 4.8% | 9.7% |
| *M. mitochondrii* | IrcNa | 85.5% | 85.5% | 0.0% | 4.8% | 9.7% |
| *“Ca.* Midichloria sp.” | Ifro | 84.7% | 84.7% | 0.0% | 4.8% | 10.5% |
| *“Ca.* Midichloria sp.” | Akure | 84.7% | 84.7% | 0.0% | 5.6% | 9.7% |
| *M. mitochondrii* | IrcESP | 84.7% | 84.7% | 0.0% | 5.6% | 9.7% |
| *M. mitochondrii* | IrcVa | 80.6% | 79.8% | 0.0% | 5.6% | 13.8% |
| *M. mitochondrii* | IrcAchnasheen | 85.5% | 85.5% | 0.0% | 4.8% | 9.7% |
| *“Ca.* Midichloria sp.” | ESP | 86.3% | 86.3% | 0.0% | 3.2% | 10.5% |
| *M. mitochondrii* | Asinara | 80.6% | 80.6% | 0.0% | 3.2% | 16.2% |
